# A framework for developing an evidence-based, comprehensive tobacco control program

**DOI:** 10.1186/1478-4505-8-17

**Published:** 2010-05-27

**Authors:** Laura Rosen, Elliot Rosenberg, Martin McKee, Shosh Gan-Noy, Diane Levin, Elana Mayshar, Galia Shacham, John Borowski, Gabi Bin Nun, Boaz Lev

**Affiliations:** 1 Dept. of Health Promotion, School of Public Health, Sackler Faculty of Medicine, Tel Aviv University and Chair, Tobacco Control Subcommittee, Healthy Israel 2020, Israel; 2 National Coordinator, Healthy Israel 2020 Initiative, Israel Ministry of Health, 33 Pierre Koenig St, Jerusalem, Israel; 3 European Centre on Health of Societies in Transition London School of Hygiene and Tropical Medicine Keppel Street London WC1E 7HT UK; 4 Dept. of Health Education and Promotion, Clalit Health Services, 101 Arlozorov St., Tel Aviv, Israel; 5 Legal Department, Israel Ministry of Health, 2 Ben Tabai St, Jerusalem, Israel; 6 Psychological Consulting Service Dept., Israel Ministry of Education, 2 Rechov Dvora, Jerusalem, Israel; 7 Emergency Department, Shaare Zedek Medical Center, 12 Bayit St., Jerusalem, Israel, 91031; 8 School of Management, Department of Health Systems Management, Ben Gurion University POB 653, Beer Sheva 84105, ISRAEL; 9 Office of the Associate Director General, Israel Ministry of Health, 2 Ben Tabai St, Jerusalem, Israel

## Abstract

**Background:**

Tobacco control is an area where the translation of evidence into policy would seem to be straightforward, given the wealth of epidemiological, behavioural and other types of research available. Yet, even here challenges exist. These include information overload, concealment of key (industry-funded) evidence, contextualization, assessment of population impact, and the changing nature of the threat.

**Methods:**

In the context of Israel's health targeting initiative, Healthy Israel 2020, we describe the steps taken to develop a comprehensive tobacco control strategy. We elaborate on the following: a) scientific issues influencing the choice of tobacco control strategies; b) organization of existing evidence of effectiveness of interventions into a manageable form, and c) consideration of relevant philosophical and political issues. We propose a framework for developing a plan and illustrate this process with a case study in Israel.

**Results:**

Broad consensus exists regarding the effectiveness of most interventions, but current recommendations differ in the emphasis they place on different strategies. Scientific challenges include integration of complex and sometimes conflicting information from authoritative sources, and lack of estimates of population impact of interventions. Philosophical and political challenges include the use of evidence-based versus innovative policymaking, the importance of individual versus governmental responsibility, and whether and how interventions should be prioritized.

The proposed framework includes: 1) compilation of a list of potential interventions 2) modification of that list based on local needs and political constraints; 3) streamlining the list by categorizing interventions into broad groupings of related interventions; together these groupings form the basis of a comprehensive plan; and 4) refinement of the plan by comparing it to existing comprehensive plans.

**Conclusions:**

Development of a comprehensive tobacco control plan is a complex endeavour, involving crucial decisions regarding intervention components. "Off the shelf" plans, which need to be adapted to local settings, are available from a variety of sources, and a multitude of individual recommendations are available. The proposed framework for adapting existing approaches to the local social and political climate may assist others planning for smoke-free societies. Additionally, this experience has implications for development of evidence-based health plans addressing other risk factors.

## Background

Controlling tobacco use, the leading preventable cause of death in the world today [1], remains an elusive goal. The combination of the addictive nature of smoking, industry activity, and tax revenue to governments complicate control attempts. Greater understanding of the enormity of the tobacco-related disease burden has highlighted the importance of controlling active and involuntary exposure.

Because individual measures have limited effectiveness, and groups of strategies implemented together seem to offer greater promise than individual strategies [2], comprehensive plans for tobacco control have gained popularity. The policy maker has no shortage of sources of advice. The US Centers for Disease Control [2,3], the WHO's Framework Convention for Tobacco Control [1] (FCTC) [1] and MPOWER [4], the US National Institutes of Health [5], and the US Institute of Medicine [6], and have been joined by individual US states [7-9], countries worldwide [10] and leading tobacco control activists [11,12] in proposing comprehensive plans. Meanwhile, evidence for the effectiveness of individual measures has been reviewed by various organizations, for example, the Cochrane Collaboration, the US Task Force for Community Preventive Services [13] the US Preventive Services Task Force [14], the US Public Health Service [15], and Britain's National Institute for Health and Clinical Excellence (NICE) [16].

Faced with these myriad sources of information, the salient question facing policy makers is: ***Which strategies and interventions should be included in the plan? ***To answer this, policy makers must address both scientific and philosophical issues in the context of the local political constraints. Recommendations with a strong evidence base may not be politically feasible. For example, subsidizing cessation services has been proven to increase cessation, yet, a society with a strong emphasis on individual responsibility may oppose such a move. Likewise, increasing the excise tax on cigarettes and earmarking percentages of the increased revenue for tobacco control activities will inevitably provide an important resource for tobacco control, but some finance ministries oppose earmarking of funds as it restricts their freedom to set budgets. Proposed measures must also be adapted to the ability of states to enforce measures.

Extensive scientific evidence and related recommendations for practice are available. These include interventions at both the individual level, as well as those geared for entire communities, for large organizations, and comprehensive programs at the national and international levels. Philosophical and political issues such as the degree of reliance on pure scientific evidence, the desirability of, and best method to prioritization interventions, and the best strategy to designate ultimate responsibility for tobacco control, have been less well addressed.

## Methods

In this paper we propose a framework for developing comprehensive tobacco control plans. We address scientific issues affecting choice of strategies and interventions; suggest a system to organize the existing evidence into a manageable form; discuss the philosophical and political issues that arise in the course of such an endeavour; and present a systematic means of adapting scientific knowledge, philosophic principles, and policy recommendations to the local setting. As a case study, we report the Israeli experience in developing a national plan relevant to local needs, aspirations, and means.

### Process of development of an Israeli comprehensive tobacco control plan

The Healthy Israel 2020 initiative was created in 2005 with a mandate to develop national health targets and recommend evidence-based interventions necessary to achieve them [17]. The Health Behaviors committee is one of its 21 committees. The Tobacco Control Subcommittee (subsequently referred to as "the Committee) is a leading subcommittee thereof. It is comprised of 24 national leaders in tobacco control, from the government, non-governmental organizations, health maintenance organizations, and academia, and is strengthened by support from international consultants. A consensus-based process was used for decision making.

From 2005-2008, the Committee met regularly to develop health objectives, set quantitative targets for each objective, and develop a set of strategies and specific interventions to achieve them. Concomitantly, members collaborated to produce a summary of the existing evidence to serve as the backbone for writing recommendations for interventions.

Initially, the leadership of Healthy Israel 2020 envisioned the following multi-stage process for selecting a set of interventions necessary to reach the health targets:

1 - Evidence-based interventions were to be selected from various sources, including the Cochrane Collaboration, the US Preventive Services Task Force, the US Task Force on Community Preventive Services, and other literature reviews and original studies, ranked by level of evidence.

2 - Interventions were to be selected from the resultant list based on current patterns of tobacco use in Israel, feasibility of implementation, and the existing political constraints.

3 - The items were to be prioritized on the basis of their potential future impact, quality of the evidence of effectiveness, and their generalizability as gleaned from the scientific literature. The information from the scientific literature was to be complimented by local evidence and the Committee's expert opinion on feasibility of implementation. Cost, cost-effectiveness, and issues of inequality were also to be considered.

The final product was to be a prioritized list of interventions.

## Results and Discussion

### Scientific issues affecting choice of strategies

Challenges in developing evidence-based tobacco control policy are summarized below.

#### Information overload

Information overload is a problem common to guideline development in many areas of medicine and public health [18]. Thousands of studies and hundreds of reviews have been published on the effectiveness of individual interventions for tobacco control, as have numerous comprehensive plans. Reviewing all of the evidence is rarely a feasible strategy for those developing plans. Yet, even a review of reviews in this field is challenging, especially as reviews may be of varying quality and produce conflicting results [19]. An alternative is to draw on authoritative sources of systematic reviews, and base recommendations on interventions proposed by leading agencies. However, these are written from various perspectives and, when prepared by national bodies, reflect their particular circumstances.

#### Accessibility of important evidence

Conventionally, public health professionals seek evidence from the published literature. However, in the case of tobacco, much research was done by the industry with the explicit intention that it not be published. Furthermore, some research that has been published was undertaken with the explicit aim of confusing the picture, as part of a process of denialism, used not only by the tobacco industry but also others in, for example, the oil and alcohol industry [20]. A wealth of material is now available from the industry's internal documents, now in the public domain following litigation in the USA: it is here that much evidence on the toxicity of second-hand smoke is to be found [21,22]. These documents are also an important source of evidence on effectiveness of policies, on industry perceptions of their effectiveness, and may provide country-specific information on industry tactics. Although an increasing amount of this material is now being published by researchers analysing these documents, there is still a great deal that has yet to be studied, and especially material that relates to the activities of the tobacco industry in individual countries.

#### Uneven quality of the evidence base

The strength of scientific evidence for interventions varies [23], and policy makers must decide when there is sufficient evidence to act. Many governments acted long before there was conclusive biological evidence that smoking caused cancer or heart disease. In retrospect, these actions did, indeed, prove prescient [24].

The situation is more complicated when deciding about specific interventions. Measures that, intuitively, seem worthwhile may turn out to be ineffective or even harmful: for example, the youth anti-smoking campaigns sponsored by the tobacco industry portrayed smoking as adult behaviour, but in doing so may have actually increased the probability of initiation [25].

The classical hierarchy of evidence [26] forming the basis of many medical decisions is primarily premised upon internal validity. Other forms of research with greater potential impact may be assigned low scores. In tobacco control, much of the effort takes place in the policy arena, where controlled trials may be difficult or impossible to implement. Thus, unrelated to actual effectiveness, the clinical interventions which are assessed with randomized controlled trials have an inherent advantage. A further problem is that external validity is often not addressed [27], so policy makers may have difficulty determining whether interventions will work in different populations and settings.

#### Population impact often unavailable

Most research papers and many systematic reviews provide estimates of effectiveness of interventions. However, in the policy setting, a point estimate of effectiveness provides insufficient information on which to base policy. The important outcome is population impact: How much will implementing this particular strategy affect tobacco use in the population as a whole?

As an example of the difference between these parameters, consider brief physician-based counselling for cessation. This has been shown to increase quit rates by 74%, corresponding to an absolute increase of 1%-3% in quit rates [28]. Yet, the population impact of such counselling remains unknown. Although it is possible to develop predictive models, the data needed to populate them are often lacking. Calculation would require knowledge of the percentage of physicians who counsel or would be willing to counsel [29] and frequency of physician visits by smokers at different ages (as the expected change in life expectancy secondary to cessation is a function of the age of quitting).

#### The need for innovation: keeping up with the tobacco industry

Notwithstanding the limitations of the available evidence, there is a clear imperative that public health policy should draw on what does exist to the extent possible. Since the tobacco industry is constantly seeking to stay one step ahead of the tobacco control community, there is a continuing need for innovation. One contemporary example is the need to address the marketing of smokeless tobacco [30,31], increasingly recognised as a tactic to maintain levels of addiction among individuals who might otherwise be weaned off nicotine in smoke-free settings [32], as well as to entice new users. For example, it has now been shown that those who use both cigarettes and oral tobacco have higher rates of nicotine addiction than those using either alone. Thus, it is important to be aware of the growing evidence of effectiveness of measures to limit the use of smokeless tobacco and of the increasing involvement of the traditional cigarette companies in the smokeless tobacco market, a situation that enables them to circumvent actual and potential marketing restrictions.

Another is action against product placement in films [33]. Although the tobacco industry has been paying actors and studios to promote its products since the 1930s, the scale on which it operated has only recently become apparent [34]. However, the prevalence of smoking in movies declined until the early 1980s, coinciding with falling rates in the general population. Since then it has increased, so that actors in movies are now much more likely to smoke than those they are portraying [35]. These findings have stimulated tobacco control researchers to ask why, revealing that cumulative exposure to smoking in movies significantly increases the probability that a young adult will smoke [36], with a subsequent randomised controlled trial finding that among young smokers exposed to smoking or non-smoking images, the former were more likely to smoke in the break after viewing the images than the latter [37]. This has led some public authorities (e.g., in England and California) to consider requiring films containing smoking scenes to have an adult rating [38]. However, even though such measures have yet to be implemented, they may already need to be adapted given evidence of the use of televised trailers to portray smoking scenes to those who may not be able to view the actual movies legally [39]. A third is the way in which the industry is circumventing measures of packaging. For example, the New Zealand government's attempts to get manufacturers to remove misleading descriptors such as "light" was undermined by the explicit association of what was portrayed as lightness or mildness with certain colours that remained in use after the words had been removed and by the use of synonyms such as "subtle" or "mellow"[40]. These and other findings on how people perceive packaging [41] provide a strong case for requiring cigarettes to be sold only in plain packages.

These examples are important because none of them are addressed in existing recommendations. Thus, those involved in tobacco control must constantly keep up to date with the tactics adopted by the tobacco industry to circumvent controls and the evidence for new approaches to control. This involves familiarity with the medical and social research literature, the industry's trade media, and research using the industry's internal documents.

### Review of the scientific evidence

#### Specific interventions

The evidence related to specific interventions is summarized in Additional File 1, Table S1, and draws on systematic reviews from the Cochrane Collaboration (4^th ^quarter 2008) [42], the US Preventive Services Task Force [14], and the Task Force on Community Preventive Services [13]. A similar review of the evidence was published in 2001, nearly a decade ago[43]. Interventions reviewed took place in clinical and community settings, and spanned legislation, enforcement, taxation, medical financing, communication, education, and clinical prevention. The three main activities were prevention of uptake (especially among youths and young adults), cessation, and prevention of exposure to second hand smoke. Effectiveness of cessation medications was recently reviewed elsewhere [15] and is not addressed here.

Tax increases, legislation and enforcement of smoking bans and restrictions, mass media campaigns, workplace interventions, and quitlines are effective strategies in community settings. In the clinical setting, screening and brief advice by health professionals, pharmacotherapy, and counselling (individual and group) are effective. Subsidizing the cost of cessation and provider reminder systems is effective at the health system level. School-based approaches have limited effectiveness.

#### Comprehensive plans

Comprehensive strategies have been proposed by authoritative bodies including the WHO (the FCTC [1], and the MPOWER program [4]), the IOM (Blueprint for the Nation on Ending the Tobacco Problem [6]), and the US CDC (Best Practices for Comprehensive Tobacco Control Programs [3]).

The plans differ in how they address the tension between evidence-based and innovative policymaking. CDC Best Practices and MPOWER were primarily based on evidence-based interventions. The FCTC is a negotiated document based on consensus of members of participating states, rather than a direct report of scientific evidence. The IOM report is two-pronged: the first prong is based on evidence, while the second is comprised of innovative techniques.

The "building blocks" of these proposed programs are summarized in Table 1. Most recommend surveillance and research, with a consensus on the need to monitor the prevalence of tobacco use. MPOWER, the FCTC, and the CDC all recommend monitoring of tobacco policy. Protecting the public from second hand smoke exposure is recommended by all, though the specific measures vary. Only the IOM explicitly recommends bans in cars carrying children, smoke-free apartment buildings, and advice by healthcare providers on the dangers of second-hand smoke.

**Table 1:** Comparison of strategies from international comprehensive tobacco control plans

	MPOWER	CDC Best Practices	Institute of Medicine	Framework Convention for Tobacco Control
**MONITORING AND RESEARCH**	√	√		√
Monitor prevalence of tobacco use	√	√		Article 20
Monitor attitudes, knowledge, or social norms		√		Article 20
Monitor tobacco industry marketing, promotion and lobbying	√			Article 20
Monitor tobacco production and manufacture				Article 20
Monitor policies, impact of policies	√	√		Article 20
Assess effectiveness and impact of programs	√	√		Article 20
Research on consequences of tobacco use and exposure		√		Article 20
Research on determinants of tobacco use and exposure	√	√		Article 20
Identification of alternative crops				Article 20
**PROTECT PEOPLE FROM SECONDHAND SMOKE**	√	√ (Details left to States)	√	√
Prohibition of smoking in all indoor non-residential environments	√		√	Article 8
Full enforcement	√			
Voluntary home smoking bans in homes with children	√		√	
Healthcare provider messages on SHS			√	
Car bans with children passengers			√	
Smoke-free apartment buildings			√	
**OFFER HELP TO QUIT (AND ENCOURAGE QUITTING)**	√	√	√	√
Medical advice	√	√	√	Article 14
Pharmacotherapy	√	√	√	Article 14
Quit lines	√	√	√	
Counselling	√	√	√	Article 14
Provider training/providing trained personnel	√	√	√	Article 12
Subsidize costs	√	√	√	Article 14
**WARN ABOUT DANGERS**	√	√	√	√
Warn about dangers	√	√	√	Article 12
Advertising campaigns about active smoking	√	√	√	
Advertising campaigns about involuntary smoking	√	√		
Warnings on packs	√		√	Article 11.
FDA authority to regulate warnings			√	
**YOUTH PREVENTION**	√	√	√	√
Funded school programs		√	√	
Media campaigns	√	√	√	
Limit youth access		√	√	Article 16
**COMMUNITY, STATE, FEDERAL, AND INTERNATIONAL ACTION**	√	√	√	√
Establishment of a coordinating body for tobacco control				Article 5
Comprehensive state tobacco control programs		√	√	
Link with chronic disease programs		√		
Identify tobacco-related disparities between population groups	√	√		
Create tobacco-free social norms		√		
Funding strategy	√	√	√	Article 26
Training and support for researchers				Article 20
Cooperate with WHO on research, report and exchange of information; Cooperation in scientific, technical, and legal fields				Article 20, 22
**REGULATION AND LITIGATION**	√	√	√	√
Raise taxes/Prices	√	√	√	Article 6
Enforced bans, bans, or restrictions on tobacco advertising, promotion, sponsorship	√	√	√	Article 13
Regulation of manufacture, distribution, marketing, and use by FDA or other regulatory agency			√	Articles 9, 15
Tobacco product disclosure			√	Article 10
Disclosure of research by industry			√	
Reduce nicotine level			√	
Support for economically viable alternative activities				Article 17
Litigation/Liability				Article 19

All of the organizations recommend helping smokers quit, using a similar set of strategies: medical advice, pharmacotherapy, quitlines, counselling, and subsidizing costs of the aforementioned interventions.

All of the organizations advocate warning the public about dangers via health communication. Enforcement or implementation of tobacco advertising bans, or bans on tobacco promotion and sponsorship, are mentioned in all of the plans, though the CDC leaves the details to individual states.

Tobacco tax increases are recommended by all organizations. The FCTC uniquely addresses tobacco supply as well as demand, and also has provisions for assisting farmers and producers of tobacco products to find alternative employment.

Far-reaching regulatory activity at the federal level is recommended by the IOM, much of it identical to the FCTC recommendations. The IOM also recommends mandatory reduction in the nicotine content of tobacco products.

Planning and administration at the state and community level are stressed by the CDC, along with the creation of a strategic plan. The FCTC recommends creation of a body with responsibility for tobacco control.

The CDC emphasizes tobacco-related disparities and supports addressing them through culturally appropriate interventions.

### Philosophical and political issues integral to the planning process

Little has been written to date regarding philosophic issues confronted by policy makers in developing comprehensive tobacco control plans. The main issues are summarized below.

#### Overarching goals: A function of societal values

The first step in the planning process is to agree on the overarching goals. While this may seem obvious at the outset, it is actually complex as it must take into account societal values and political preferences, and should be consistent with broader health policies. Thus, a policy designed to reduce the overall tobacco-attributable disease burden may conflict with one seeking to reduce health disparities: some interventions may be most effective among groups who are relatively privileged, so that average health improves but disparities widen. A goal of reducing current prevalence of tobacco use will lead to somewhat different priorities than one of reducing future preventable tobacco-related disease. Policies should be aligned with stated goals.

#### Individual or governmental responsibility?

Decisions made by policy makers implicitly reflect philosophical beliefs about the balance of governmental and individual responsibility for health. This is a subject rife with inconsistencies [44].

A decision to develop a comprehensive plan presupposes a role for government in tackling the harm caused by tobacco, a principle enshrined in the FCTC and one which most states have ratified. However, it cannot be assumed that this view is held *de facto *by decision-makers. Their preferences are rarely explicit, but may be inferred from the nature of existing policies.

Many WHO members still have much to do to implement the provisions of the FCTC. Obstacles include ideological opposition to state action in areas that can be portrayed, even if incorrectly, as personal choice, the strength of corporate interests, and a failure to balance tax revenues from cigarettes against the cost of smoking-related disease. Even in countries which are loathe to regulate industry or individual behaviour, there is often consensus on the need to protect citizens from second-hand smoke. Thus, many American cities and states have passed smoke-free air laws. The State of New York goes beyond this, currently providing free nicotine replacement therapy to smokers.

For these reasons it will often be necessary to explicitly address beliefs about the relationship between individuals, corporations, and governments that underlie any plan, addressing the question of what elements of tobacco control are legitimate areas for government action [45]. In principle, individuals should be allowed to make their own decisions about how they lead their lives, as long as those decisions meet certain criteria. First, all costs and benefits should be private, affecting no-one else. This is clearly not the case with tobacco, because of the direct harm caused by second-hand smoke and the societal costs resulting from the reduced productivity of smokers who are unwell or die prematurely. Second, the decision should be rational, maximising their utility by weighing costs and benefits. The empirical lack of rationality in decisions made by children and the problem of nicotine addiction in adolescence [46] provides a justification for action to limit smoking in this age group. Third, individuals should have accurate, up-to-date information, and not be affected by "optimism bias" which would lead them to believe that they are at less at risk than others [47]. Again, there is substantial evidence that both the risk to health and the addictive nature of tobacco are underestimated [48]. Fourth, individuals should be time-consistent, balancing long-term harms against instant gratification. In reality, the latter is given much greater weight than the former. For all of these reasons, there is a powerful case for government action, but the argument must be made clearly.

Yet another reason for developing a consistent set of principles is to prevent manipulation by the tobacco industry. The industry and its front organizations will focus attention on any inconsistencies, while invoking the "slippery slope" argument to portray any attempt to control smoking as the first step towards either a "nanny state" or a totalitarian regime controlling all aspects of life [49]. Clarity about principles is also helpful in gaining support for individual policies that, although effective, may pose difficulties for some politicians. In this context, there is a strong case for advertising that explicitly counters arguments made by the tobacco industry and sheds light upon tactics used to subvert effective health policies [50].

#### Prevention, cessation, or reduction of secondhand smoke exposure?

Prevention, cessation, and reduction of second-hand smoke exposure are all essential elements of a comprehensive plan. Changes in any of these three elements impacts the others: prevention of uptake and smoking cessation lead to reduced second-hand smoke exposure; interventions which reduce second-hand smoke exposure also reduce prevalence of smoking (in part because they denormalize the act of smoking and make life more difficult for smokers) [51], and prevention of smoking initiation obviates the need for cessation interventions.

Deciding what to prioritize involves philosophical and scientific considerations. Those who believe that the individual bears sole or primary responsibility for smoking will be less likely to advocate government expenditure on cessation medications and other aids. Protection of non-smokers in public places may be more acceptable to these fierce individualists.

Advocates of prevention focus on the advantage of intervening before addiction occurs. Advocates of cessation point out the quicker impact on disease burden that cessation will bring, due to the long lag time for the development of many smoking-related illnesses [52], as well as the immediate benefits of cessation for smokers [53]. Cessation proponents also point to the limited effectiveness of many prevention strategies. A further issue is age. When prevention is the objective, youth tend to be targeted; on the other hand, cessation efforts generally focus on adults. Even among adults, a case can be made for differential targeting, as younger adults have more to gain than older adults [54]. Yet withholding cessation aids from older adults is ageist.

#### Can evidence of effectiveness of interventions from other countries be generalized to a local setting?

A central question is whether information about effectiveness of interventions is transferrable between countries. Social norms and political climates differ substantially, so a strategy which is feasible or effective in one country or region or society may be impossible to implement or ineffective in another setting. Much of the available evidence is produced in the developed nations, which have the resources to both deliver the interventions and assess their effectiveness.

Some of the evidence base is built on research performed in a variety of countries, such as that on the effects of smoke-free air laws. Yet, even that literature is mostly limited to research performed in Western nations, and generalization to countries with deficient public compliance to governmental regulation is problematic.

Hence, transferring evidence which can be found in the literature from other countries, societies, or cultures may not represent the effectiveness or feasibility of an intervention in another place or time. On the other hand, evidence is likely to be sparse from smaller or less developed countries. Dismissing evidence, and in particular, robust, multi-national evidence simply because it comes from elsewhere, would not benefit countries without the resources to perform their own research.

#### Prioritized list versus comprehensive programme

Policy makers must decide whether to recommend a comprehensive package or a prioritized list of interventions. Some public health bodies, such as the Partnership for Prevention, produce prioritized lists of preventive interventions under the assumption that policy makers seek such guidance to maximize the impact of their limited health budgets. Current recommendations from organizations reviewed here recommend comprehensive policies as opposed to prioritized lists, largely because of mounting evidence that interventions work better in combination than separately [3]. However, a recent review of implementation of the CDC Best Practices [55] is instructive: It showed that most state-run tobacco control programs do not receive the minimum levels of funding recommended by the CDC. States have been forced to decide between implementing only some of the interventions, or allocating partial funding for each element. So, prioritization inevitably takes place, albeit at a later stage. Policy makers should consider the possibility of explicit prioritization at the outset. Alternatively, policy makers might strategically prioritize to optimize their effectiveness by time, that is, by deciding which interventions would best be done first. For example, it may be worthwhile to first build popular support for legislation through investing in mass media approaches, and only afterwards endeavour to pass legislation.

### Case Study: Development of a national tobacco control plan within the context of Healthy Israel 2020

#### Actual process

The actual process used by the Committee was substantially modified during the course of deliberations. The first change came about because some Committee members were interested in submitting proposals for innovative interventions, which did not have the advantage of supporting evidence. On the one hand, this went against the grain of a truly evidence-based plan; on the other, innovation in tobacco control is crucial to counter the continually-changing tactics of the tobacco industry. So it was decided to allow innovative interventions. Like the evidence-based interventions, these had to undergo approval by the full Committee. Further, the innovative nature of these interventions was duly noted, in the context of the transparent rankings of the evidence base which were provided for all interventions.

A further issue which arose was the fact that there was little local evidence regarding effectiveness of interventions. We found and used what little local evidence we could. For the most part, however, we were forced to choose between using the available evidence, even though it came from outside of Israel, or having almost no evidence to use. We chose the former, but considered local cultural, social, and political norms when making our plan.

The most significant change to the program occurred when we reached what was to be the final stage of the work: prioritization of the various interventions. Like all the Healthy Israel 2020 Committees, we were tasked with not simply providing a list of interventions, but with providing a prioritized list of interventions. The details of how to prioritize were to be determined by the Committee and based on a combination of potential future impact on disease burden, quality of the evidence of effectiveness, generalizability, equity, cost, cost-effectiveness, and feasibility.

As we began to consider different options for prioritization, we were confronted with three serious problems:

i. We could not find a single measure on which to prioritize which was acceptable to the committee. Balancing the needs of future population impact, equity, strength of evidence, and cost did not lend itself to any kind of straightforward prioritization; further, estimates for most of these measures were not easily available, and estimation was beyond the scope of the 2020 initiative.

ii. The list which we had developed included 76 interventions. This was unwieldy and also had many interventions which were similar.

iii. As we delved farther into the literature on tobacco control policy and comprehensive tobacco control plans, we found evidence that the most effective policies were not based on single strategies or prioritized lists of interventions, but on multi-component plans in which the different components complimented one another.

We grappled with these three issues in a full Committee meeting. In a breakthrough decision, we decided to move away from the concept of a long, prioritized list of interventions and, instead, adopted the concept of a comprehensive plan based on a much smaller number of grouped interventions. These broad categorizations would include multiple individually-related items, but would be unprioritized: the idea was that they would be recommended as a set of strategies instead of as a prioritized list. We considered categorizations based on various factors (for example: setting (community, school, clinical) or targeted outcome (prevention, cessation, second hand smoke)); before deciding on the groupings listed below.

Once we had created the main strategic groupings, we sorted the individual interventions into the groups and then compared our categories with existing comprehensive plans. We made some revisions on the basis of these existing plans before finalizing our plan.

**Figure 1:**
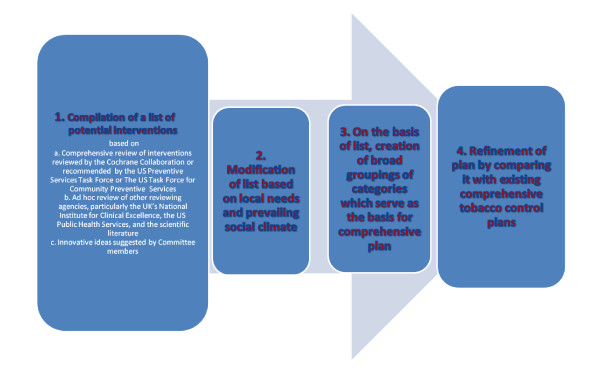
Framework for developing a comprehensive tobacco control program

The final framework for developing the plan is illustrated in Figure 1. The process had four distinct stages:

1 - Compilation of a list of potential interventions based on

• Comprehensive review of interventions reviewed by the Cochrane Collaboration, and/or recommended by the US Preventive Services Task Forces, and the US Task Force for Community Preventive Services.

• Ad hoc review of interventions examined by other reviewing agencies, particularly the UK's National Institute for Clinical Excellence, The US Public Health Service Clinical practice guideline: Treating Tobacco Use and Dependence: 2008 Update, and the scientific literature

• Innovative ideas suggested by Committee members.

2 - Modification of that list based on local needs and the prevailing political climate

We qualitatively considered relevance to the Israeli situation, practicability, the current political climate, and potential population impact (to the extent possible). Exclusions were primarily based on lack of political feasibility. Thus, for example, legislating decreased nicotine content was not considered feasible, as most Israeli cigarettes are imported. While a call for increased excise taxes was included, a call for earmarking part of that income for tobacco control activities was not, due to the known opposition of the Treasury to earmarking of funds.

3 - On the basis of the list, creation of broad groupings of interventional strategies to form the basis of a comprehensive plan

The broad categories were to cover all the main elements of tobacco control. In many cases, similar strategies are important for different goals. For example, mass media is important for prevention of smoking initiation, cessation, and reduction of second hand smoke exposure. Constructing the categories was influenced by the existing evidence for effectiveness. School interventions, for example, were not defined as a separate category because of the dearth of evidence for effectiveness from these interventions. Interventions were categorized into eight broad strategies (Taxation, legislation, enforcement, promotion and support for smoking cessation, mass media, community interventions, surveillance, research), to be coordinated by a central organizing body. Because of the politically sensitive nature of the structure of the central organizing body, we did not include a recommendation for the structure of that body.

4 - Refinement of the plan by comparing it to existing comprehensive plans

We added a category for monitoring which included monitoring of tobacco industry activities and monitoring of effects of implemented interventions. Neither of those elements had previously been included in the plan.

#### The comprehensive national tobacco control plan

The final set of strategies and interventions, representing a comprehensive plan for national tobacco control, is presented in Table 2, with supporting evidence of effectiveness in Additional File 2, Table S2.

**Table 2:** Strategies and interventions recommended by the Tobacco Control 2020 Committee (Summary version)

**ESTABLISHMENT OF A CENTRAL BODY**	A central body, with appropriate authority and budget, for coordinating and implementing strategies for reducing tobacco use and exposure to second hand smoke (SHS) including the recommendations of the Healthy Israel 2020 subcommittee, and fulfilling FCTC obligations.
**TAXATION OF TOBACCO PRODUCTS AND USE OF REGULATORY MECHANISMS**	Increase in taxation on all tobacco products, imports and local.
	Preventing exposure to SHS Absolute ban on smoking in public places including workplaces; Ban on smoking in special open spaces like public swimming pools, beaches, bus stops and train stations; Ban on smoking inside private motor vehicles where minors are present.
	Advertising prohibition/Disclosure of ingredients/Preventing conflict of interest in research
**LEGISLATION**	Prohibition of advertising tobacco products using any form of media; Prohibition of tobacco industry sponsorship; prohibition of point of sale advertising, together with an obligation to display a color poster outlining health damages from smoking at point of sale; Packaging to be labelled with large graphic warnings including information on smoking cessation; Obligatory disclosure by tobacco companies of all ingredients, and substances released upon lighting tobacco products including information on toxicology of said substances; Obligatory testing of tobacco products by the local authorities funded by the tobacco companies as per FCTC recommendations; Restriction of Academic Bodies from receiving sponsorship or research grants from tobacco companies.
	Restriction on Tobacco Sales Prohibition of tobacco sales from automatic vending machines; Prohibition on sales of tobacco products via the internet or in shops exempted from full taxation (duty free) and in any other way without full taxation.
**ENFORCEMENT**	Effective enforcement of all tobacco related legislation including limitation of SHS exposure prohibition of sales to minors, advertising, warning labels, illegal trade.
**PROMOTION AND SUPPORT FOR SMOKING CESSATION**	Telephone hotline for smoking cessation counselling and support; Smoking cessation workshops; Individual smoking cessation counselling; Screening of smoking status of all patients by physician or other medical staff and recording in the patient's file; Brief counselling by physician, nurse or medical team members, use of ABC counselling method; Use of over-the-counter (OTC) Nicotine Replacement Therapy (NRT) or doctor-prescribed pharmacological agents for smoking cessation; Medication in addition to counselling; Counselling in addition to medication; Intensive (8 session) counselling in addition to medication; Counselling adolescents; Focusing of advice on smoking cessation on the pregnant population; Training of health care teams about smoking cessation counselling therapy; Subsidizing ("basket of health services") proven pharmacological and non-pharmacological treatments for smoking cessation; Development and distribution of materials for "self help" smoking cessation; School based smoking cessation programs
**MASS MEDIA**	Combination of mass media campaigns with other tobacco control efforts (legislation, community programs, schools etc); Improving the public's knowledge of how mass media influence attitudes to smoking; Prohibition of advertising or sponsorship by tobacco companies, including point of sale advertising; Establishment of an internet based support service; Changing the media's depiction of a "smoking world" by minimizing images of smoking celebrities, etc; Decreasing the presentation of smokers (especially of cigarettes) in movies, television, and the theatre;
**COMMUNITY INTERVENTIONS**	Smoking cessation interventions in the workplace; Implementation of "Smoke Free Schools" policy; School based programs for smoking prevention; Implementation of "Smoke free Israel Defense Forces (IDF)" policy; Program for parents and teachers to prevent children's exposure to SHS.
**SURVEILLANCE**	Development of national surveillance systems, including Surveillance of population smoking behaviour; Participation in the "Global Youth Surveillance System" or another similar system advocated by the WHO, which includes surveys of students, teachers and schools regarding smoking and related activities; Surveillance of exposure to secondhand smoke, knowledge and attitudes towards smoking according to WHO guidelines; Monitoring of governmental actions on tobacco control policy (including Knesset and other activities); Monitoring and reporting of monies received by the government from tobacco sales or other activities; Monitoring of governmental and HMO expenditures due to tobacco use; Monitoring of tobacco industry activities to promote tobacco use; Monitoring tobacco product content (nicotine and other substances)
**RESEARCH**	Identification or development of successful interventions for prevention of use of tobacco products (especially among youth), cessation for smokers (including adolescent smokers), and prevention of exposure to secondhand smoke (especially among pregnant women, infants, and children). Particular emphasis on the use of social marketing techniques and workplace interventions; Research on the economic and health costs of tobacco in Israel; Research on the health damages caused by smoking; Research into the economic determinants of tobacco use.

A summary of the Healthy Israel 2020 Committee's report was presented to the Knesset (Israeli Parliament) as part of the Health Minister's 2008 report on smoking. The plan has an impact on tobacco control activities of the Ministries of Health and Education. It is expected to gain cachet when the full Healthy Israel 2020 Initiative report is published later this year.

## Conclusions

Development of a comprehensive tobacco control plan is a complex endeavour, involving crucial decisions regarding intervention components. "Off the shelf" plans, which need to be adapted to local settings, are available from a variety of sources, and a multitude of individual recommendations are available. The proposed framework is based on the following steps: 1: compilation of a list of potential interventions based on current evidence, augmented with innovative suggestions; 2: modification of that list based on local needs, philosophical principles, and the prevailing political climate; 3: streamlining the list by categorizing interventions into broad groupings of related interventions; together these groupings form the basis of a comprehensive plan, and 4: refinement of the plan by comparing it to existing comprehensive plans.

This framework may assist others planning for smoke-free societies. Additionally, this experience has implications for development of evidence-based health plans addressing other risk factors.

## List of abbreviations

**CDC**: Centers for Disease Control (US); **FCTC**: Framework Convention for Tobacco Control; **HMO**: Health Maintenance Organization; **IOM**: Institute of Medicine (US); **NICE**: National Institute for Clinical Excellence (UK); **NIH**: National Institute of Health (US); **USPSTF**: US Preventive Services Task Force; **WHO**: World Health Organization; USPHS: US Public Health Service

## Competing interests

The authors declare that they have no competing interests.

## Authors' contributions

LJR is Chair of the Healthy Israel 2020 Tobacco Control Subcommittee. She initiated this paper and wrote the initial draft. MM contributed to the writing. ER and JB assisted in preparing the tables and additional files. All authors participated in the process of developing the plan, critically edited the paper, and reviewed the final manuscript.

## Supplementary Material

Additional file 1 **Table S1**. Tobacco control interventions reviewed by the Cochrane Collaboration, the US Preventive Services Task Force, and the Task Force for Community Preventive Services Click here for file

Additional file 2 **Table S2**. Strategies and interventions recommended by the Tobacco Control 2020 Committee (Explicit evidence base included) Click here for file
