# Supplementary material for: A framework for developing an evidence-based, comprehensive tobacco control program
Source: Health Res Policy Syst. 2010 May 27;8:17. doi: 10.1186/1478-4505-8-17 (PMC2894826; doi:10.1186/1478-4505-8-17)
Supplement: Additional file 1 — Table S1. Tobacco control interventions reviewed by the Cochrane Collaboration, the US Preventive Services Task Force, and the Task Force for Community Preventive Services [file 1478-4505-8-17-S1.DOC]

**Additional File 1** **(Table S1)** Tobacco control interventions reviewed by the Cochrane Collaboration, the US Preventive Services Task Force, and the Task Force for Community Preventive Services

| Details | USPSTF ^[1]^  2008 | Community Guide ^[2]^  Published 2005 | Cochrane ^[3]^  4^th^ quarter 2008 |
| --- | --- | --- | --- |
| **Clinical interventions to identify and treat tobacco use and dependence** | | | |
| Screen all adult patients for tobacco use and provide cessation interventions : Brief cessation counseling interventions, including screening, brief counseling (3 minutes or less), and/or pharmacotherapy; ABC behavioral counseling framework; screening systems to identify and document tobacco use; FDA approved pharmacotherapy | Strongly recommended: A |  |  |
| Brief counseling by physicians (<=3 minutes) | (As above) |  | Effective  *Brief advice vs. none: RR 1.66 [1.42,1.94] Gives absolute difference of 1-3% in cessation rate. Intense vs. minimal: RR 1.37, 95% CI (1.20 to 1.56).* ^[4]^ |
| Advice and assistance by nurses | (As above) |  | Effective  *OR: 1.28, [ 1.18,1.38]* ^[5]^ |
| Advice by dental professionals |  |  | Effective  *OR: 1.44 [1.16,1.78]* ^[6]^ |
| Pharmacotherapy*  (This report does not cover names of specific medications) | Strongly recommended: A |  | Effective.  *Specific medications are covered in individual reviews but are not summarized here.* |
| Acupuncture, acupressure, laser therapy, electrical stimulation |  |  | Overall, no consistent evidence for effectiveness  *Acupuncture vs. sham, short term effect: OR=1.36 [1.07,1.72]* ^[7]^ |
| Aversive smoking |  |  | Overall: insufficient evidence.  *Rapid smoking vs. control: OR=2.01 [1.36,2.95]* ^[8]^ |
| Biomedical risk assessment |  |  | Insufficient evidence. ^[9]^ |
| Hypnotherapy |  |  | Insufficient evidence. ^[10]^ |
| Screen all pregnant women + provide counseling | Strongly recommended: A |  |  |
| Interventions for promoting cessation during pregnancy |  |  | Effective.  *Intervention reduction: RR 0.94 [0.93, 0.95], absolute difference of 6 per 100 women continuing to smoke. Low birthweight RR .81 [0.70,0.94], preterm birth RR .84 [0.72-0.98], increase in birth weight 33g [11, 55]*. ^[11]^ |
| Screening and counseling children and adolescents | Insufficient Evidence |  |  |
| Telephone support with possible other components(self help, NRT, counseling, groups) |  | Recommended  Increase quit rates by 3 per 100 | Effective.  *Quit rates higher for groups randomized to receive multiple sessions of call back counseling OR 1.41 [1.27,1.57]. Counselling not initiated by calls to hotlines OR: 1.33 [1.21,1.47]* ^[12]^ |
| Preoperative Smoking cessation |  |  | Effective  *No pooled effect estimated due to heterogeneity*  ^[13]^ |
| Hospitalized patients cessation |  |  | Effective ( 1 month )  *Intensive intervention: Control: OR: 1.65 [1.44,1.90]. Less intensive NS*. ^[14]^ |
| **system wide interventions** | | | |
| Provider reminder systems (alone) |  | Recommended  *Increase quit rates by 4 additional per 100; increase clients receiving advice by 13 additional per 100; increasing screening by 32 additional clients per 100* |  |
| Provider reminder systems with or without client education |  | Recommended  Increasing clients who quit by 5 additional clients per 100. Additional 20 clients per 100 received advice to quit. |  |
| Provider education systems (alone) |  | Insufficient evidence | No strong evidence for increased quit rates among patients  *Smoker identification increased, and providers 1.5 -2.5 times more likely to intervene* ^[15]^ |
| Provider feedback |  | Insufficient evidence |  |
| Reduce costs to patient for cessation |  | Recommended  *Increase clients who successfully quit by 8 clients per 100. Increase use of cessation therapies by additional 7 per 100.* | Effective  *Increase of 2% [0, 0.05] abstinence.*  *Full coverage versus none) OR: 1.48 [1.17,1.88]*  *Full coverage versus partial OR: 2.49 [1.59,3.90].* ^[16]^ |
| **Community interventions to reduce exposure to secondhand smoke** | | | |
| Smoking bans and restrictions |  | Recommended  *Decrease amount of environmental tobacco smoke (chemical components) by 72%, exposure by 60%* | Effective: *Complete bans with strong management support*  Ineffective: *Signs alone*  Helpful: I*ntensive educational campaigns and multi-component strategies.*  *Narrative review, no OR available* ^[17]^ |
| Community / family and caregiver education to reduce environmental tobacco smoke in the home |  | Insufficient evidence | Limited evidence: *Intensive counseling intervention*  *Narrative summary, no OR available* ^[18]^ |
| **Community interventions to reduce initiation by children and adolescents, and to reduce youth access** | | | |
| Increase prices |  | Recommended  *10% price increase results in 4% decrease in consumption; among adolescents and young adults, 10% price increase results in 2% decrease in prevalence* |  |
| Mass media in combinations to prevent or reduce initiation in young people |  | Recommended  *Decrease number of young people using tobacco by 2.4%; better in campaigns lasting more than two years* | Some (not strong) evidence ^[19]^ |
| Community interventions or mobilization to prevent smoking in young people |  | Recommended  *Decrease in tobacco use in students by 5.8%*, reduce sale to youths by 34% | Some limited support ^[20]^  *Narrative review.* |
| Restrictions on youth access |  |  | Effective: *Illegal sales reduced,* *effect not sustained**.*  Limited evidence: *Effect on youth perception of ease of access to tobacco and smoking behavior.*  *Narrative review, no OR available*^[21]^ |
| Sales laws directed at retailers when implemented alone |  | Insufficient evidence |  |
| Laws directed at minors’ purchase, possession, or use, when implemented alone |  | Insufficient evidence |  |
| Enforcement of retailer restriction laws, when implemented alone |  | Insufficient evidence | See above ^[21]^ |
| Retailer education with reinforcement + health info |  | Insufficient evidence |  |
| Retailer education without reinforcement, when implemented alone |  | Insufficient evidence | Less effective: *Education alone*  ^[21]^ |
| Community education about minors’ access when implemented alone |  | Insufficient evidence |  |
| Family-based programmes |  |  | Insufficient evidence *Mixed results, related in study quality* ^[22]^ |
| School based programs |  |  | Some evidence: *Short term effectiveness.*  Little evidence: *Information alone is effective.*  No evidence: *Long term effectiveness.*  *Narrative review , no OR in abstract* ^[23]^ |
| **Community interventions to increase cessation [and reduce prevalence]** | | | |
| Community interventions to reduce prevalence |  |  | Not effective. *[-1%-+3%] decline.*  *Best studies failed to detect effect.* ^[24]^ |
| Increase unit price |  | Recommended  *10% price increase causes 4% decrease in consumption, 2% decrease in prevalence* |  |
| Mass media education campaigns in combination (tax increases, community wide) |  | Recommended  *Additional 2 quitters per 100; 12.8% reduction in consumption; reduce prevalence of tobacco use by 3 people per 100 tobacco users* |  |
| Mass media campaigns to encourage cessation among adults |  |  | Effective  *No summary statistic available.* ^[25]^ |
| Mass media education – cessation series |  | Insufficient evidence |  |
| Mass media education -cessation contests |  | Insufficient evidence | Quit and Win Contests: Increased quit rates but population impact small  *No meta-analysis. 3/5 studies saw higher quit rates (8-20%) in intervention group. Less than 1/500 smokers quit due to contest. Deception high*. ^[26]^ |
| Competitions and incentives (workplaces) |  |  | Not Effective. *Some early success, but benefit dissipated when reward not offered.* ^[27]^ |
| Community pharmacy personnel interventions for cessation |  |  | Limited evidence  *Narrative approach, no OR available* ^[28]^ |
| Enhancing partner support |  |  | Not effective  *6-9 months OR: 1.01 [.86,1.18]*  *12 months OR: 1.04 [.87,1.24]* ^[29]^ |
| Exercise |  |  | Insufficient evidence  *1 of 13 trials provided evidence for effectiveness*  *Narrative approach, no OR available* ^[30]^ |
| Group behavior therapy |  |  | Effective  *Group versus self help: OR 2.04 [1.6, 2.6] Group versuss placebo: OR 2.17 [1.37,3.45]* ^[31]^ |
| Self-help |  |  | Effective  *Effect small. Self help vs. none: OR: 1.24 [[1.07,1.45] – after exclusion of 2 positive trials which produced heterogeneity. Tailored: OR: 1.42 [1.26,1.61]* ^[32]^ |
| Telephone support with possible other components(self help, NRT, counseling, groups) (See above: Clinical interventions to identify and treat tobacco dependence) |  | Increase quit rate by 3 smokers per 100 | Effective. *Quit rates higher for groups randomized to receive multiple sessions of call back counseling OR 1.41 [1.27,1.57]. Counseling not initiated by calls to hotlines OR: 1.33 [1.21,1.47]* ^[12]^ |
| Cessation for young people | Insufficient evidence |  | Insufficient evidence ^[33]^ |
| Individual behavioural counselling |  |  | Effective  *Individual vs. control OR: 1.39 [1.24,1.57]. In trials with NRT as well OR:1.27 [1.02,1.59].* ^[34]^ |
| Behavioral and pharmacological treatments for smokeless tobacco use cessation |  |  | Pharmacological: *No effects on longterm abstinence.*  Behavioral: *Recommended. Effects seen in some studies.* ^[35]^ |
| Relapse prevention |  |  | Insufficient evidence ^[36]^ |
| Workplace |  | Decreased smoking prevalence by 15% | Effective ^[38]^  *Narrative review due to heterogeneity*  *Individual counselling, group counselling or use of NRT are equally effective, while organization-wide interventions such as contests, incentives, or comprehensive programs are not effective* |
| **OTHER interventions** | | | |
| Reducing use of waterpipes |  |  | No trials ^[39]^ |
| Harm reduction |  |  | Effective: *Short-term reduction in number of cigarettes per day with NRT: OR 2.02 [1.55, 2.62].*  Insufficient evidence: *Longterm reduction, longterm health benefit of reduction unclear.* ^[40]^ |
| Tobacco advertising |  |  | Effective: *Increasing smoking among adolescents. No OR available.* ^[41]^ |

***Note:*** *Following each recommendation are codes indicating the sources of supporting evidence proving the effectiveness of the recommendation. Level of evidence is indicated in parenthesis after the letter indicating the source. Example: C(A-B) indicates that Cochrane found the effect to be statistically significant. P(A) the USPSTF strongly recommended the intervention****.***

***Sources of evidence and levels of recommendations or decision regarding evidence of effectiveness:***

*C = Cochrane Collaboration: A or B: Effective****,*** *I: Insufficient Evidence****Note:*** *Cochrane presents quantitative or qualitative summaries of the evidence. When quantitative summaries are used, the Odds Ratio (OR) followed by the Confidence Interval is presented.*

*P = United States Preventive Services Task Force (USPSTF) (In use at the time tobacco recommendations were made (i.e., prior to 2007):A: Strongly Recommended, B: Recommended, C: No recommendation, D: Not Recommended, I: Insufficient Evidence to Make a Recommendation*

*G = Task Force on Community Preventive Services ("Guide"): A: Recommended (Strong evidence of effectiveness)****,*** *B: Recommended**(Sufficient evidence of effectiveness), I: Insufficient Evidence*

*T = USPHS - US Department of Health and Human Services-* *Public Health Service Clinical practice guideline: Treating Tobacco Use and Dependence: 2008 Update.*

*A = Multiple well-designed randomized clinical trials, directly relevant to the recommendation, yielded a consistent pattern of findings B = Some evidence from randomized clinical trials supported the recommendation, but the scientific support was not optimal. For instance, few randomized trials existed, the trials that did exist were somewhat inconsistent, or the trials were not directly relevant to the recommendation. C = Reserved for important clinical situations in which the Panel achieved consensus on the recommendation in the absence of relevant randomized controlled trials.*

*F=* ***F****ramework Convention on Tobacco Control*

*OSR =* ***O****ther* ***S****ystematic* ***R****eview*

*OE =* ***O****ther* ***E****vidence (not systematic review, not necessarily interventional)*

*LE =* ***L****ocal (Israeli)* ***E****vidence*

*EO =* ***E****xpert* ***O****pinion*

***Key to evidence ranking:***

*1= Effective (A-B) as ranked by Cochrane or strongly recommended (A) by the USPSTF, the "Guide", or the USPHS*

*2= Recommended (B) by the USPSTF or the "Guide", by a national or international panel such as the IOM of FCTC, or in an interventional study in a peer-reviewed scientific publication*

*3=Expert opinion*

WEB APPENDIX REFERENCES

1. **US Dept of Health and Human Services, Agency for Healthcare Research and Quality. The Guide to Clinical Preventive Services 2008: Recommendations of the US Preventive Services Task Force. AHRQ Pub. No. 08-05122 September 2008 ISBN No. 978-1-58763-359-1**

2. Zaza S, Briss P, Harris K: ***The Guide to Community Preventive Services: What Works to Promote Health?***: Oxford University Press; 2005.

3. **The Cochrane Collaboration Home Page. Internet site: http://www.cochrane.org/. Accessed Aug. 5, 2009.**

4. Stead LF, Bergson G, Lancaster T: **Physician advice for smoking cessation**. *Cochrane Database of Systematic Reviews* 2008, **Issue 2. Art. No.: CD000165. DOI: 10.1002/14651858.CD000165.pub3. Last assessed as up-to-date: 13 February 2008.**
5. Rice VH, Stead LF: **Nursing interventions for smoking cessation**. *Cochrane Database of Systematic Reviews* 2008, **Issue 1. Art. No.: CD001188. DOI: 10.1002/14651858.CD001188.pub3. Last assessed as up-to-date: 23 January 2008.**

6. Carr AB, Ebbert JO: **Interventions for tobacco cessation in the dental setting. Cochrane Database of Systematic Reviews**. *Cochrane Database of Systematic Reviews* 2006, **Issue 1. Art. No.: CD005084. DOI: 10.1002/14651858.CD005084.pub2. Last assessed as up-to-date: 25 January 2006.**

7. White AR, Rampes H, Campbell J: **Acupuncture and related interventions for smoking cessation.,**. *Cochrane Database of Systematic Reviews* 2006, **Issue 1. Art. No.: CD000009. DOI: 10.1002/14651858.CD000009.pub2. Last assessed as up-to-date: Oct 23, 2005.**

8. Hajek P, F. SL: **Aversive smoking for smoking cessation. Cochrane Database of Systematic Reviews**. *Cochrane Database of Systematic Reviews* 2001, **Issue 3. Art. No.: CD000546. DOI: 10.1002/14651858.CD000546.pub2. Last assessed as up-to-date: Jan 29, 2007.**

9. Bize R, Burnand B, Mueller Y, Cornuz J: **Biomedical risk assessment as an aid for smoking cessation** *Cochrane Database of Systematic Reviews* 2005, **Issue 4. Art. No.: CD004705. DOI: 10.1002/14651858.CD004705.pub2. First published: Oct. 19, 2005**

10. Abbot NC, Stead LF, Whitr AR, Barnes J: **Hypnotherapy for smoking cessation**. *Cochrane Database of Systematic Reviews* 1998, **ssue 2. Art. No.: CD001008. DOI: 10.1002/14651858.CD001008. Last assessed as up-to-date: Feb 15, 2005.**

11. Lumley J, Oliver SS, Chambelain C, Oakley L: **Interventions for promoting smoking cessation during pregnancy**. *Cochrane Database of Systematic Reviews* 2004, **Issue 4. Art. No.: CD001055. DOI: 10.1002/14651858.CD001055.pub2. Last assessed as up-to-date: 18 October 2004.**

12. Stead LF, Perera R, Lancaster T: **Telephone counselling for smoking cessation**. *Cochrane Database of Systematic Reviews* 2006, **Issue 3. Art. No.: CD002850. DOI: 10.1002/14651858.CD002850.pub2. Last assessed as up-to-date: 10 April 2006.**

13. Muller A, Villebro N: **Interventions for preoperative smoking cessation**. *Cochrane Database of Systematic Reviews* 2005, **Issue 3. Art. No.: CD002294. DOI: 10.1002/14651858.CD002294.pub2.Last assessed as up-to-date: May 17, 2005.**

14. Rigotti N, Munfano MR, Stead LF: **Interventions for smoking cessation in hospitalised patients**. *Cochrane Database of Systematic Reviews* 2007, **Issue 3. Art. No.: CD001837. DOI: 10.1002/14651858.CD001837.pub2. Last assessed as up-to-date: 19 May 2007**.

15. Lancaster T, Fowler G: **Training health professionals in smoking cessation**. *Cochrane Database of Systematic Reviews* 2000, **Issue 3. Art. No.: CD000214. DOI: 10.1002/14651858.CD000214. Last assessed as up-to-date: 30 May 2000.**

16. Kaper J, Wagena EJ, Severens JL, Van Schayck CP: **Healthcare financing systems for increasing the use of tobacco dependence treatment**. *Cochrane Database of Systematic Reviews* 2005, **Issue 1. Art. No.: CD004305. DOI: 10.1002/14651858.CD004305.pub2. Most recent amendment: Nov. 16, 2004.**

17. Serra C, Bonfill X., Pladevaill-Vila M: **Interventions for preventing tobacco smoking in public places**. *Cochrane Database of Systematic Reviews* 2008, **Issue 3. Art. No.: CD001294. DOI: 10.1002/14651858.CD001294.pub2. Last assessed as up-to-date: 19 March 2006.**

18. Priest N, Roseby R, Waters E, Polnay A, Campbell R, Spencer N, Webster P, Ferguson-Thorne G: **Family and carer smoking control programmes for reducing children's exposure to environmental tobacco smoke**. *Cochrane Database of Systematic Reviews* 2008, **Issue 4. Art. No.: CD001746. DOI: 10.1002/14651858.CD001746.pub2. Last assessed as up-to-date: 7 August 2008.**

19. Sowden AJ, L. A: **Mass media interventions for preventing smoking in young people. Cochrane Database of Systematic Reviews,** . *Cochrane Database of Systematic Reviews* 1998, **Issue 4. Art. No.: CD001006. DOI: 10.1002/14651858.CD001006. Last assessed as up-to-date: 19 October 1999.**

20. Sowden AJ, Stead LF: **Community interventions for preventing smoking in young people**. *Cochrane Database of Systematic Reviews* 2003, **Issue 1. Art. No.: CD001291. DOI: 10.1002/14651858.CD001291.Last assessed as up-to-date: Sept. 23, 2002.**

21. Stead LF, Lancaster T: **Interventions for preventing tobacco sales to minors**. *Cochrane Database of Systematic Reviews* 2005, **Issue 1. Art. No.: CD001497. DOI: 10.1002/14651858.CD001497.pub2. Last assessed as up-to-date: Apr 30, 2008.**

22. Thomas RE, Baker PRA, Lorenzetti D: **Family-based programmes for preventing smoking by children and adolescents**. *Cochrane Database of Systematic Reviews* 2007, **Issue 1. Art. No.: CD004493. DOI: 10.1002/14651858.CD004493.pub2. Last assessed as up-to-date: Dec. 15, 2007.**

23. Thomas RE, Perera R: **School-based programmes for preventing smoking**. *Cochrane Database of Systematic Reviews* 2006, **Issue 3. Art. No.: CD001293. DOI: 10.1002/14651858.CD001293.pub2. Last assessed as up-to-date: 19 April 2006.**

24. Secker-Walker R, Gnich W, Platt S, Lancaster T: **Community interventions for reducing smoking among adults**. *Cochrane Database of Systematic Reviews* 2002, **Issue 2. Art. No.: CD001745. DOI: 10.1002/14651858.CD001745. Last assessed as up-to-date: Jan 30, 2006.**

25. Bala M, Strzeszynski L, Cahill K: **Mass media interventions for smoking cessation in adults**. *Cochrane Database of Systematic Reviews* 2008, **Issue 1. Art. No.: CD004704. DOI: 10.1002/14651858.CD004704.pub2. Last assessed as up-to-date: 11 November 2007.**

26.Hey K, Perera R: **Quit and Win contests for smoking cessation**. *Cochrane Database of Systematic Reviews* 2007, **Issue 4. Update Feb 17, 2005**.

27. Hey K, Perera R: **Competitions and incentives for smoking cessation**. *Cochrane Database of Systematic Reviews* 2007, **Issue 4. Update: Feb 18, 2005**(4).

28. Sinclair HK, Bond CM, Stead LF: **Community pharmacy personnel interventions for smoking cessation**. *Cochrane Database of Systematic Reviews* 2004, **Issue 1. Art. No.: CD003698. DOI: 10.1002/14651858.CD003698.pub2.Last assessed as up-to-date: Oct. 30, 2008.**

29. Park EW, Schultz JK, Tudiver FG, Campbell T, Becker LA: **Enhancing partner support to improve smoking cessation**. *Cochrane Database of Systematic Reviews* 2004, **Issue 3. Art.No.:CD002928. DOI: 10.1002/14651858.CD002928.pub2. Last assessed as up-to-date: Feb. 24, 2008.**

30. Ussher MH, Taylor A, Faulkner G: **Exercise interventions for smoking cessation**. *Cochrane Database of Systematic Reviews* 2008, **Issue 4. Art. No.: CD002295. DOI: 10.1002/14651858.CD002295.pub3. Last assessed as up-to-date: July 5,2008.**

31. Stead LF, Lancaster T: **Group behaviour therapy programmes for smoking cessation**. *Cochrane Database of Systematic Reviews* 2005, **Issue 2. Art. No.: CD001007. DOI: 10.1002/14651858.CD001007.pub2. Date most recent amendment: Update: Feb 16, 2005.**

32. Lancaster T, Stead LF: **Self-help interventions for smoking cessation**. *Cochrane Database of Systematic Reviews* 2005, **Issue 3. Art. No.: CD001118. DOI: 10.1002/14651858.CD001118.pub2. Last assessed as up-to-date: 20 July 2005.**

33. Grimshaw GM, Stanton A: **Tobacco cessation interventions for young people**. *Cochrane Database of Systematic Reviews* 2006, **Issue 4. Art. No.: CD003289. DOI: 10.1002/14651858.CD003289.pub4. Last assessed as up-to-date: 18 October 2006.**

34. Lancaster T, Stead L: **Individual behavioural counselling for smoking cessation**. *Cochrane Database of Systematic Reviews* 2005, **Issue 2. Art. No.: CD001292. DOI: 10.1002/14651858.CD001292.pub2.Last assessed as up-to-date. Jul 14, 2008.**

35. EEbbert JO, Montori V, Vickers KS, Erwin PC, Dale LC, Stead LF: **Interventions for smokeless tobacco use cessation. Cochrane Database of Systematic Reviews**. *Cochrane Database of Systematic Reviews* 2007, **Issue 4. Art. No.: CD004306. DOI: 10.1002/14651858.CD004306.pub3. Last assessed as up-to-date: 17 October 2007.**

36. Hajek P, Stead LF, West R, Jarvis M, Lancaster T: **Relapse prevention interventions for smoking cessation**. *Cochrane Database of Systematic Reviews* 2005, **Issue 1. Art. No.: CD003999. DOI: 10.1002/14651858.CD003999.pub2 Last assessed as up-to-date: 24 January 2005.**

37. **Guide to Community Preventive Services. Decreasing tobacco use in worksite settings: incentives and competitions to increase smoking cessation among workers. www.thecommunityguide.org/tobacco/worksite/incentives.html <http://www.thecommunityguide.org/tobacco/worksite/incentives.html>. Accessed: July 16, 2009**

38. Moher M, Hey K, Lancaster T: **Workplace interventions for smoking cessation**. *Cochrane Database of Systematic Reviews* 2008, **Issue 4. Last assesed as Up-to-Date: Apr 23, 2008**.

39. Mazaik W, Ward K. D., Eissenberg T: **Interventions for waterpipe smoking cessation**. *Cochrane Database of Systematic Reviews* 2007, **Issue 4. Art. No.: CD005549. DOI: 10.1002/14651858.CD005549.pub2. Last assessed as up-to-date: 8 August 2007**.

40. Stead LF, Lancaster T: **Interventions to reduce harm from continued tobacco use**. *Cochrane Database of Systematic Reviews* 2007, **Issue 3. Art. No.: CD005231. DOI: 10.1002/14651858.CD005231.pub2 . Last assessed as up-to-date: 18 July 2007.**

41. Lovato C, Linn G, Stead LF, Best A: **Impact of tobacco advertising and promotion on increasing adolescent smoking behaviours**. *Cochrane Database of Systematic Reviews* 2003, **Issue 4. Art. No.: CD003439. DOI: 10.1002/14651858.CD003439.Last assessed as up-to-date: May 12, 2003.**

42. Repace J: **Benefits of smoke-free regulations in outdoor settings: beaches, golf courses, parks, patios, and in motor vehicles**. *William Mitchell Law Review* 2008:1621-1638.

43. Diethelm P, Rielle JC, McKee M: **The whole truth and nothing but the truth? The research that Phillip Morris doesn’t want you to see**. *Lancet* 2005, **366**:86-92.

44. Fiore MC, Jaיn CR, Baker TB, al. e: *Treating Tobacco Use and Dependence: 2008 Update. Clinical Practice Guideline. May 2008*. Rockville, MD: : U.S. Department of Health and Human Services. Public Health Service ; 2008.

45. **NICE  Guidelines on Smoking Cessation, Brief interventions and referral for smoking cessation in primary care and other settings. 3/2006. Australia - NSW Dep. Of Health 2005. www.nice.org.uk/page.aspx?o=SmokingCessationMain** [www.nice.org.uk/page.aspx?o=SmokingCessationMain]

46. Walters S, Wright J, Shegog R: **A review of computer and Internet-based interventions for smoking behavior**. *Addictive Behaviors* 2006, **31**(2):264-277.

47. Sargent J, Beach M, Adachi-Mejia A, Gibson J, Titus-Ernstoff L, Carusi C, et al.: **Exposure to Movie Smoking: Its Relation to Smoking Initiation Among US Adolescents.** *Pediatrics* 2005, **116**(5):1183-1191.

48. Tickle JJ, Sargent JD, Dalton MA, Beach ML, Heatherton TF: **Favourite movie stars, their tobacco use in contemporary movies, and its association with adolescent smoking.** *British Medical Journal* 2001, **10**:16.

49. Sargent J, Dalton M, Beach M: **Exposure to cigarette promotions and smoking uptake in adolescents: Evidence of a dose-response relation**. *Tob Control* 2000, **9**:163.

50. **National Cancer Institute. The Role of the Media in Promoting and Reducing Tobacco Use. Tobacco Control Monograph No. 19.Bethesda, MD:U.S. Department of Health and Human Services, National Institutes of Health, National Cancer Institute, NIH Pub.No. 07-6242, June 2008.**

51. Fichtenberg C, Glantz S: **Effect of smoke-free workplaces on smoking behavior: systematic review**. *British Medical Journal* 2002, **325**:188.

52. Aveyard P, Markham WA, Cheng KK: **A methodological and substantive review of the evidence that schools cause pupils to smoke**. *Social Science & Medicine* 2004, **58**(11):2253-2265.

53. Rosen L, Verbov G, Amitai Y, Stein-Zamir C, Knishkowy B: **Reaching Jewish Ultra-Orthodox Adolescents: Results from a Targeted Smoking Prevention Trial**. In *14th World Conference on Tobacco OR Health*. Mumbai; 2009.

54. Levine H, Borowski J, Bar-Zeev Y, Shreir E, Zarka S: **Smoking Prevention- What can a primary physician accomplish in his unit?** . *Journal of Israeli Military Medicine* 2007, **4**:202-204 (Hebrew)

55. **WHO Report on the Global Tobacco Epidemic, 2008. Fresh and Alive: MPOWER. Internet site: http://www.who.int/tobacco/mpower/mpower_report_full_2008.pdf.**
