# Supplementary material for: A framework for developing an evidence-based, comprehensive tobacco control program
Source: Health Res Policy Syst. 2010 May 27;8:17. doi: 10.1186/1478-4505-8-17 (PMC2894826; doi:10.1186/1478-4505-8-17)
Supplement: Additional file 2 — Table S2. Strategies and interventions recommended by the Tobacco Control 2020 Committee (Explicit evidence base included) [file 1478-4505-8-17-S2.DOC]

**Additional File 2 (Table S2).** Strategies and interventions recommended by the Tobacco Control 2020 Committee (Detailed evidence base with rankings included)

| ***EVIDENCE BASE*** | | ***INTERVENTIONS & STRATEGIES*** |
| --- | --- | --- |
| **TAXATION OF TOBACCO PRODUCTS AND USE OF REGULATORY MECHANISMS** | | |
| ***Guide:*** Price increases recommended. 10% price increase results in 4% decrease in consumption. ^[2]^ | Increase in taxation on all tobacco products, imports and local. ^G(B)1^ | |
| **LEGISLATION** | | |
| *Preventing exposure to environmental tobacco smoke* | | |
| ***Cochrane:*** Effective: Complete bans with strong management support. ^[17]^  Effective. Smoke-free workplace interventions – effective (Narrative review; no OR). ^[38]^  ***Guide:*** Recommended. Decrease amount of environmental tobacco smoke (chemical components) by 72%, exposure by 60%. ^[2]^ | Absolute ban on smoking in public places including workplaces. ^C(A-B), G(B)1^ | |
| *Institute of Medicine:Recommends that parents make cars and homes smoke-free*  ***Other Evidence*** *^[42]^* | Ban on smoking inside private motor vehicles where minors are present. ^OE, EO2^ | |
| ***Other Evidence*** *^[42]^* | Ban on smoking in special open spaces like public swimming pools, beaches, bus stops and train stations. ^OE3^ | |
| *Advertising prohibition/Disclosure of ingredients/Preventing conflict of interest in research* | | |
| ***Cochrane:*** Effective: Tobacco advertising is effective at increasing smoking prevalence (No OR available). ^[41]^  FCTC Article 13  Each Party shall, in accordance with its constitution or constitutional principles, undertake a comprehensive ban of all tobacco advertising, promotion and sponsorship. | Prohibition of advertising tobacco products using any form of media; Prohibition of tobacco industry sponsorship, prohibition of point of sale advertising, together with an obligation to display a color poster outlining health damages from smoking at point of sale. ^C(A-B), F2^  (See also mass media article.) | |
| ***FCTC Article 11***  Each unit packet and package of tobacco products and any outside packaging and labelling of such products also carry health warnings describing the harmful effects of tobacco use, and may include other appropriate messages. These warnings and messages:  (i) shall be approved by the competent national authority,  (ii) shall be rotating,  (iii) shall be large, clear, visible and legible,  (iv) should be 50% or more of the principal display areas but shall be no less than 30% of the principal display areas,  (v) may be in the form of or include pictures or pictograms. | Packaging will be labelled with large graphic warnings including information on smoking cessation. ^F2^ | |
| ***FCTC Article 10***  Each Party shall, in accordance with its national law, adopt and implement effective legislative, executive, administrative or other measures requiring manufacturers and importers of tobacco products to disclose to governmental authorities information about the contents and emissions of tobacco products. Each Party shall further adopt and implement effective measures for public disclosure of information about the toxic constituents of the tobacco products and the emissions that they may produce. | Obligatory disclosure by tobacco companies of all ingredients, and substances released upon lighting tobacco products including information on toxicology of said substances. ^F2^ | |
| ***FCTC Article 9***  The Conference of the Parties, in consultation with competent international bodies, shall propose guidelines for testing and measuring the contents and emissions of tobacco products, and for the regulation of these contents and emissions. | Obligatory testing of tobacco products by the local authorities funded by the tobacco companies as per FCTC recommendations. ^F2^ | |
| Tobacco sponsored research published selectively to provide advantage to industry ^[43]^ | Restriction of Academic Bodies from receiving sponsorship or research grants from tobacco companies.^OE3^ | |
| *Restriction on Tobacco Sales* | | |
| ***FCTC Article 16***  (d) ensuring that tobacco vending machines under its jurisdiction are not accessible to minors and do not promote the sale of tobacco products to minors. | Prohibition of tobacco sales from automatic vending machines. ^F2^ | |
| ***FCTC Article 6***  (b) prohibiting or restricting, as appropriate, sales to and/or importations by international travellers of tax- and duty-free tobacco products. | Prohibition on sales of tobacco products via the internet or in shops exempted from full taxation (duty free) and in any other way without full taxation. ^F2^ | |
| **ENFORCEMENT** | | |
| ***Cochrane:*** Insufficient evidence. ^[21]^  ***Guide***: Insufficient evidence.  ***FCTC Article 8***  Actively promote at other jurisdictional levels the adoption and implementation of effective legislative, executive, administrative and/or other measures, providing for protection from exposure to tobacco smoke in indoor workplaces, public transport, indoor public places. | | Effective enforcement of all tobacco related legislation including limitation of environmental tobacco smoke exposure, prohibition of sales to minors**,** ^C(I), G (I)^**^2^,** advertising, warning labels, illegal trade**.** ^F^**^2^** |
| **PROMOTION AND SUPPORT FOR SMOKING CESSATION** | | |
| ***Cochrane:*** Effective. Quit rates higher for groups randomized to receive multiple sessions of call back counseling OR 1.41 [1.27,1.57]. Counselling not initiated by calls to hotlines OR: 1.33 [1.21,1.47] ^[12]^  ***Guide:*** Recommended. Increase quit rates by 3 per 100  ***USPHS:*** OR = 1.6 [1.4, 1.8]. Estimated abstinence rate: 12.7 [11.3, 14.2] vs. 8.5 for regular care (minimal to no counselling or self-help). Telephone counselling in combination with pharmacological therapy significantly improves the quit rate compared with pharmacotherapy alone. OR = 1.3 [1.1, 1.6] Estimated abstinence rates 28.1 [24.5, 32] versus 23.2 for pharmacotherapy alone. ^[43]^ | | Telephone hotline for smoking cessation counselling and support. ^C (A-B), G(R), T(A)1^ |
| ***USPSTF:*** Screening in primary care settings strongly recommended. ^[1]^  ***OSR: NICE***: Recording recommended. ^[45]^ | | Screening smoking status of all patients by physician or other medical staff ^P(A) 1^ and recording in the patients file. ^OSR2^ |
| ***Cochrane****:* Effective.  Physician brief advice vs. none: OR 1.66 [1.42, 1.94] Gives absolute difference of 1%-3% in cessation rate. ^[4]^  Intense vs. minimal: OR: 1.37 [1.20,1.56] ^[4]^  Nurse advice effective: OR: 1.28, [ 1.18,1.38] ^[5]^  ***USPSTF***: Screening, provide brief behavioral counseling (less than 3 minutes), and pharmacotherapy in primary care settings strongly recommended ^[1]^  ***OSR:*** NICE ^[45]^ | | Brief counselling by physician, ^P (A )^, ^C(A-B)1^, nurse or medical team members.^OSR2^ |
| *Cochrane, USPSTF, USPHS, and NICE*: Some medications are effective  Recommendations regarding specific medications were beyond the mandate of this Committee. | | Use of over-the-counter (OTC) nicotine replacement therapy (NRT) ^OSR, T(B)2^ or doctor-prescribed pharmacological agents for smoking cessation. ^P(A),C(A-B), T(A)1^ |
| ***USPHS***: Recommends dual modalities if practical and acceptable to patient.  Counseling and medication vs. counselling alone: OR = 1.7 (1.3, 2.1). Estimated abstinence rate: 22.1 (18.1, 26.8) vs. 14.6 for counselling alone. ^[43]^  Counseling and medication vs. medication alone: OR= 1.4 [1.2,1.6], Estimated abstinence rate: 27.6 [25.0, 30.3] vs. 21.7 for medication alone. ^[43]^ | | Medication in addition to counselling ^T(A)1^ or counselling in addition to medication ^T(A)1^ |
| ***USPHS****:* OR= 1.7 [1.3, 2.2], Estimated abstinence rate: 32.5 [27.3, 38.3] vs. 21.8 for medication + up to 1 counselling session. ^[43]^ | | Intensive (8 session) counselling in addition to medication ^T(A)1^ |
| ***Cochrane:*** Effective. Intervention reduction: RR 0.94 [0.93, 0.95], absolute difference of 6 per 100 women continuing to smoke. Low birthweight RR .81 [0.70,0.94], preterm birth RR .84 [0.72-0.98], increase in birth weight 33g [11, 55]. ^[11]^  ***USPSTF:*** 5-15 minute counselling by clinicians strongly recommended. ^[1]^  ***USPHS****:* Psychosocial interventions: OR = 1.8 [1.4, 2.3]. Estimated abstinence rate: 13.3 [9.0, 19.4] vs. 7.6 for regular care. Self help: OR = 1.9 (1.2, 2.9). Estimated abstinence rate: 15.0 [10.1, 21.6] vs. 8.6 for regular care. ^[43]^ | | Focus advice on smoking cessation on the pregnant population. ^C(A-B), P(A), T(A)1^ |
| ***Cochrane:*** Effective. Group versus self help: OR 2.04 [1.6, 2.6] Group vs placebo: OR 2.17 [1.37,3.45] ^[31]^  ***USPHS:*** Group counselling vs. no format: OR=1.3 [1.1-1.6] | | Smoking cessation workshops ^C(A-B)1^ |
| ***Cochrane:*** Effective. Individual vs control OR: 1.39 [1.24,1.57]. In trials with NRT as well OR:1.27 [1.02,1.59]. ^[34]^  ***USPHS:*** Individual counseling vs. no format: OR=1.7 [1.4,2.0] | | Individual smoking cessation counselling. ^C(A-B)1^ |
| ***USPHS:*** OR = 1.8 (1.1, 3.0). Estimated abstinence rate: 11.6 [7.5, 17.5] vs. 6.7 for regular care. ^[43]^  *Note: Pharmacotherapy is not recommended for adolescents.* | | Counselling adolescents ^T(B)2^ |
| ***Cochrane:*** No strong evidence for increased quit rates among patients  Smoker identification increased, and providers 1.5 -2.5 times more likely to intervene ^[15]^  ***Guide:*** Insufficient evidence  ***USPHS***: OR = 2.0 (1.2, 3.4). Estimated abstinence rate: 12.0 [7.6, 18.6] vs. 6.4 for regular care. ^[43]^  ***FCTC Article 12***  (d) Effective and appropriate training or sensitization and awareness programmes on tobacco control addressed to persons such as health workers. | | Train health care teams about smoking cessation counselling therapy. ^C(I), G(I) ,T(B), F^**^2^** |
| ***Cochrane***: Effective. Increase of 2% [0, 0.05] abstinence: Full coverage (vs. none) OR: 1.48 [1.17,1.88], Full:partial OR: 2.49 [1.59,3.90] ^[16]^  ***Guide:*** Recommended. Increase clients who successfully quit by 8 clients per 100. Increase use of cessation therapies by additional 7.8 per 100.  ***USPHS***: Full coverage (vs. none) OR= 1.6 (1.2, 2.2). Estimated abstinence rate: 10.5 [8.1, 13.5] vs. 6.7 for no coverage. ^[43]^  ***FCTC Article 14***  …collaborate with other Parties to facilitate accessibility and affordability for treatment of tobacco dependence including pharmaceutical products pursuant to Article 22. Such products and their constituents may include medicines, products used to administer medicines and diagnostics when appropriate. | | Subsidize (“basket of health services”) proven pharmacological and non-pharmacological treatments for smoking cessation. ^C(A-B), G(B), T(A), F1^ |
| ***Cochrane:*** Effective. Self help vs. none: OR: 1.24 [[1.07,1.45] – after exclusion of 2 positive trials which produced heterogeneity. . Tailored: OR: 1.42 [1.26,1.61] ^[32]^  ***USPHS:*** Self-help vs. no format: OR: 1.2 [1.02-1.3] | | Development and distribution of materials for “self help” smoking cessation. ^C(A-B)1^ |
| **mass media** | | |
| ***Cochrane*** (prevention, mass media alone): Some (not strong) evidence; OR not available ^[19]^  ***Cochrane***  (cessation, mass media alone): Can be effective(Narrative review; OR not available) ^[25]^  ***Guide*** (prevention, mass media in combination): Decrease number of young people using tobacco by 2.4%; better in campaigns lasting more than two years;  ***Guide*** (cessation, mass media in combination): Recommended. Additional 2 quitters per 100; 12.8% reduction in consumption; reduce prevalence of tobacco use by 3 people per100 tobacco users.  ***FCTC Article 12***  Each Party shall promote and strengthen public awareness of tobacco control issues, using all available communication tools, as appropriate. | | Combination of mass media campaigns with other tobacco control efforts (legislation, community programs, schools etc) , ^C(B), G(A) F1^ |
| ***Expert Opinion.*** | | Improve the public’s knowledge of how mass media influence attitudes to smoking. ^EO3^ |
| ***Cochrane:*** Effective: Tobacco advertising is effective at increasing smoking prevalence (No OR available).^41^  ***FCTC Article 13***  Each Party shall, in accordance with its constitution or constitutional principles, undertake a comprehensive ban of all tobacco advertising, promotion and sponsorship.  *See also clause under “legislation.”* | | Prohibit advertising or sponsorship by tobacco companies, including point of sale advertising. ^C(A-B), F2^ |
| ***USPHS****:* Promising, but not proven. ^[43]^  ***OSR:*** Mixed results. ^[46]^ | | Establishment of an internet based support service. ^T(B-C) OSR(I)3^ |
| ***Expert Opinion*** | | Change the media portrayal of smoking by minimizing images of smoking celebrities ^EO3^ |
| ***FCTC Article 12***  Each Party shall promote and strengthen public awareness of tobacco control issues…Toward this end, each Party shall…promote (d) effective and appropriate training or sensitization and awareness programmes on tobacco control addressed to such persons as …media professionals. | | Change the positive media image of smoking of smoking (especially cigarettes). Decrease media images of famous people smoking.^F2^ |
| ***Other Systematic Review:***  OR (highest vs. lowest quartile of exposure to movies) = 2.7 (1.7, 4.2) (Evidence does not come from intervention studies.) ^[46]^  ***Other evidence***: ^[47, 48, 49]^ | | Decrease presentation of smokers (especially of cigarettes) in movies, television, and the theatre. ^OSR, OE3^ |
| **Community Interventions** | | |
| ***Cochrane:*** Narrative review (no OR). Individual counselling, group counselling or use of NRT are equally effective, while organization-wide interventions such as contests, incentives, or comprehensive programs are not effective. ^[38]^  ***Guide:*** Sufficient evidence. A combination of wide range of interventions; worksite bans, individual, group and telephone counselling; health education; social networking; (also competitions and financial incentives played minor role) decreased smoking prevalence to by 15%. ^[37]^  ***Other Systematic Review:*** Worksite bans: Reduce prevalence of smoking by 3.9%, reduce consumption by 29% ^[51]^ | | Smoking cessation interventions in the workplace. ^C(A-B) G(B)^ ^OSR1^ |
| ***Other Systematic Review:*** *Effective, no odds ratio*. ^[52]^  ***Local Evidence***: Effective. RR for smoking everywhere: 7.8 versus total smoking ban. ^[53]^ | | Implementation of “Smoke Free Schools” policy. ^OSR, LE(E)2^ |
| ***Cochrane:*** Insufficient evidence ^[23]^ | | School-based smoking cessation programs ^C(I)^ |
| ***Cochrane:***  Some evidence: Short term effectiveness.  Little evidence: information alone is effective.  No evidence: Long term effectiveness. ^[23]^ | | School based programs for smoking prevention. ^C(I long term effects)2^ |
| ***Local Evidence***: Effective. No increase in prevalence of smoking between entry and end of basic training. ^[54]^ | | Implementation of “Smoke Free Israel Defense Forces (IDF)” Policy.^LE2^ |
| ***Cochrane:*** Limited evidence: *Intensive counseling intervention* ^[18]^  ***Guide:*** Insufficient evidence | | Program for parents and teachers to prevent children’s exposure to environmental tobacco smoke. ^G(I),C(I)3^ |
| **SURVEILLANCE** | | |
| ***USPHS****:* Monitoring activities should be a part of national quality assurance programs. Routine data collection should include outcomes measurements of tobacco cessation activities. ^[43]^  ***FCTC Article 20.2***  The Parties shall establish, as appropriate, programmes for national, regional and global surveillance of the magnitude, patterns, determinants and consequences of tobacco consumption and exposure to tobacco smoke. Towards this end, the Parties should integrate tobacco surveillance programmes into national, regional and global health surveillance programmes so that data are comparable and can be analysed at the regional and international levels, as appropriate.  ***MPOWER:*** Prevalence of tobacco use; impact of policy interventions; and tobacco industry marketing, promotion and lobbying. ^[55]^ | | Development and support of national surveillance systems integrated into international surveillance programmes. ^T(C), F2^ |
| Specifics of Surveillance Programme. | | |
| Surveillance of population smoking behaviour and exposure to secondhand smoke, knowledge and attitudes towards smoking | | |
| Population testing of biomarkers for secondhand smoke exposure | | |
| Participation in the “Global Youth Surveillance System” or another similar system advocated by the WHO, which includes surveys of students, teachers and schools regarding smoking and related activities. | | |
| Monitoring of governmental actions on tobacco control policy (including Knesset and other activities) | | |
| Monitoring and reporting of monies received by the government from tobacco sales or other activities | | |
| Monitoring of governmental and HMO expenditures due to tobacco use | | |
| Monitoring of tobacco industry activities to promote tobacco use | | |
| Tobacco product content (nicotine and other substances) | | |
| **RESEARCH** | | |
| Identification or development of successful interventions for prevention of use of tobacco products (especially among youth), cessation for smokers (including adolescent smokers), and prevention of exposure to secondhand smoke (especially among pregnant women, infants, and children). Particular emphasis will be given to use of social marketing techniques and workplace interventions. | | |
| Research into the health costs caused by tobacco products and exposure to environmental tobacco smoke. | | |
| Research into the economic costs of tobacco in Israel. | | |

***Note:*** *Following each recommendation are codes indicating the sources of supporting evidence proving the effectiveness of the recommendation. Level of evidence is indicated in parenthesis after the letter indicating the source. Example: C(A-B) indicates that Cochrane found the effect to be statistically significant. P(A) the USPSTF strongly recommended the intervention****.***

***Sources of evidence and levels of recommendations or decision regarding evidence of effectiveness:***

*C = Cochrane Collaboration: A or B: Effective****,*** *I: Insufficient Evidence****Note:*** *Cochrane presents quantitative or qualitative summaries of the evidence. When quantitative summaries are used, the Odds Ratio (OR) followed by the Confidence Interval is presented.*

*P = United States Preventive Services Task Force (USPSTF) (In use at the time tobacco recommendations were made (i.e., prior to 2007):A: Strongly Recommended, B: Recommended, C: No recommendation, D: Not Recommended, I: Insufficient Evidence to Make a Recommendation*

*G = Task Force on Community Preventive Services ("Guide"): A: Recommended (Strong evidence of effectiveness)****,*** *B: Recommended**(Sufficient evidence of effectiveness), I: Insufficient Evidence*

*T = USPHS - US Department of Health and Human Services-* *Public Health Service Clinical practice guideline: Treating Tobacco Use and Dependence: 2008 Update.*

*A = Multiple well-designed randomized clinical trials, directly relevant to the recommendation, yielded a consistent pattern of findings B = Some evidence from randomized clinical trials supported the recommendation, but the scientific support was not optimal. For instance, few randomized trials existed, the trials that did exist were somewhat inconsistent, or the trials were not directly relevant to the recommendation. C = Reserved for important clinical situations in which the Panel achieved consensus on the recommendation in the absence of relevant randomized controlled trials.*

*F=* ***F****ramework Convention on Tobacco Control*

*OSR =* ***O****ther* ***S****ystematic* ***R****eview*

*OE =* ***O****ther* ***E****vidence (not systematic review, not necessarily interventional)*

*LE =* ***L****ocal (Israeli)* ***E****vidence*

*EO =* ***E****xpert* ***O****pinion*

***Key to evidence ranking:***

*1= Effective (A-B) as ranked by Cochrane or strongly recommended (A) by the USPSTF, the "Guide", or the USPHS*

*2= Recommended (B) by the USPSTF or the "Guide", by a national or international panel such as the IOM of FCTC, or in an interventional study in a peer-reviewed scientific publication*

*3=Expert opinion*

WEB APPENDIX REFERENCES

1. **US Dept of Health and Human Services, Agency for Healthcare Research and Quality. The Guide to Clinical Preventive Services 2008: Recommendations of the US Preventive Services Task Force. AHRQ Pub. No. 08-05122 September 2008 ISBN No. 978-1-58763-359-1**

2. Zaza S, Briss P, Harris K: ***The Guide to Community Preventive Services: What Works to Promote Health?***: Oxford University Press; 2005.

3. **The Cochrane Collaboration Home Page. Internet site: http://www.cochrane.org/. Accessed Aug. 5, 2009.**

4. Stead LF, Bergson G, Lancaster T: **Physician advice for smoking cessation**. *Cochrane Database of Systematic Reviews* 2008, **Issue 2. Art. No.: CD000165. DOI: 10.1002/14651858.CD000165.pub3. Last assessed as up-to-date: 13 February 2008.**
5. Rice VH, Stead LF: **Nursing interventions for smoking cessation**. *Cochrane Database of Systematic Reviews* 2008, **Issue 1. Art. No.: CD001188. DOI: 10.1002/14651858.CD001188.pub3. Last assessed as up-to-date: 23 January 2008.**

6. Carr AB, Ebbert JO: **Interventions for tobacco cessation in the dental setting. Cochrane Database of Systematic Reviews**. *Cochrane Database of Systematic Reviews* 2006, **Issue 1. Art. No.: CD005084. DOI: 10.1002/14651858.CD005084.pub2. Last assessed as up-to-date: 25 January 2006.**

7. White AR, Rampes H, Campbell J: **Acupuncture and related interventions for smoking cessation.,**. *Cochrane Database of Systematic Reviews* 2006, **Issue 1. Art. No.: CD000009. DOI: 10.1002/14651858.CD000009.pub2. Last assessed as up-to-date: Oct 23, 2005.**

8. Hajek P, F. SL: **Aversive smoking for smoking cessation. Cochrane Database of Systematic Reviews**. *Cochrane Database of Systematic Reviews* 2001, **Issue 3. Art. No.: CD000546. DOI: 10.1002/14651858.CD000546.pub2. Last assessed as up-to-date: Jan 29, 2007.**

9. Bize R, Burnand B, Mueller Y, Cornuz J: **Biomedical risk assessment as an aid for smoking cessation** *Cochrane Database of Systematic Reviews* 2005, **Issue 4. Art. No.: CD004705. DOI: 10.1002/14651858.CD004705.pub2. First published: Oct. 19, 2005**

10. Abbot NC, Stead LF, Whitr AR, Barnes J: **Hypnotherapy for smoking cessation**. *Cochrane Database of Systematic Reviews* 1998, **ssue 2. Art. No.: CD001008. DOI: 10.1002/14651858.CD001008. Last assessed as up-to-date: Feb 15, 2005.**

11. Lumley J, Oliver SS, Chambelain C, Oakley L: **Interventions for promoting smoking cessation during pregnancy**. *Cochrane Database of Systematic Reviews* 2004, **Issue 4. Art. No.: CD001055. DOI: 10.1002/14651858.CD001055.pub2. Last assessed as up-to-date: 18 October 2004.**

12. Stead LF, Perera R, Lancaster T: **Telephone counselling for smoking cessation**. *Cochrane Database of Systematic Reviews* 2006, **Issue 3. Art. No.: CD002850. DOI: 10.1002/14651858.CD002850.pub2. Last assessed as up-to-date: 10 April 2006.**

13. Muller A, Villebro N: **Interventions for preoperative smoking cessation**. *Cochrane Database of Systematic Reviews* 2005, **Issue 3. Art. No.: CD002294. DOI: 10.1002/14651858.CD002294.pub2.Last assessed as up-to-date: May 17, 2005.**

14. Rigotti N, Munfano MR, Stead LF: **Interventions for smoking cessation in hospitalised patients**. *Cochrane Database of Systematic Reviews* 2007, **Issue 3. Art. No.: CD001837. DOI: 10.1002/14651858.CD001837.pub2. Last assessed as up-to-date: 19 May 2007**.

15. Lancaster T, Fowler G: **Training health professionals in smoking cessation**. *Cochrane Database of Systematic Reviews* 2000, **Issue 3. Art. No.: CD000214. DOI: 10.1002/14651858.CD000214. Last assessed as up-to-date: 30 May 2000.**

16. Kaper J, Wagena EJ, Severens JL, Van Schayck CP: **Healthcare financing systems for increasing the use of tobacco dependence treatment**. *Cochrane Database of Systematic Reviews* 2005, **Issue 1. Art. No.: CD004305. DOI: 10.1002/14651858.CD004305.pub2. Most recent amendment: Nov. 16, 2004.**

17. Serra C, Bonfill X., Pladevaill-Vila M: **Interventions for preventing tobacco smoking in public places**. *Cochrane Database of Systematic Reviews* 2008, **Issue 3. Art. No.: CD001294. DOI: 10.1002/14651858.CD001294.pub2. Last assessed as up-to-date: 19 March 2006.**

18. Priest N, Roseby R, Waters E, Polnay A, Campbell R, Spencer N, Webster P, Ferguson-Thorne G: **Family and carer smoking control programmes for reducing children's exposure to environmental tobacco smoke**. *Cochrane Database of Systematic Reviews* 2008, **Issue 4. Art. No.: CD001746. DOI: 10.1002/14651858.CD001746.pub2. Last assessed as up-to-date: 7 August 2008.**

19. Sowden AJ, L. A: **Mass media interventions for preventing smoking in young people. Cochrane Database of Systematic Reviews,** . *Cochrane Database of Systematic Reviews* 1998, **Issue 4. Art. No.: CD001006. DOI: 10.1002/14651858.CD001006. Last assessed as up-to-date: 19 October 1999.**

20. Sowden AJ, Stead LF: **Community interventions for preventing smoking in young people**. *Cochrane Database of Systematic Reviews* 2003, **Issue 1. Art. No.: CD001291. DOI: 10.1002/14651858.CD001291.Last assessed as up-to-date: Sept. 23, 2002.**

21. Stead LF, Lancaster T: **Interventions for preventing tobacco sales to minors**. *Cochrane Database of Systematic Reviews* 2005, **Issue 1. Art. No.: CD001497. DOI: 10.1002/14651858.CD001497.pub2. Last assessed as up-to-date: Apr 30, 2008.**

22. Thomas RE, Baker PRA, Lorenzetti D: **Family-based programmes for preventing smoking by children and adolescents**. *Cochrane Database of Systematic Reviews* 2007, **Issue 1. Art. No.: CD004493. DOI: 10.1002/14651858.CD004493.pub2. Last assessed as up-to-date: Dec. 15, 2007.**

23. Thomas RE, Perera R: **School-based programmes for preventing smoking**. *Cochrane Database of Systematic Reviews* 2006, **Issue 3. Art. No.: CD001293. DOI: 10.1002/14651858.CD001293.pub2. Last assessed as up-to-date: 19 April 2006.**

24. Secker-Walker R, Gnich W, Platt S, Lancaster T: **Community interventions for reducing smoking among adults**. *Cochrane Database of Systematic Reviews* 2002, **Issue 2. Art. No.: CD001745. DOI: 10.1002/14651858.CD001745. Last assessed as up-to-date: Jan 30, 2006.**

25. Bala M, Strzeszynski L, Cahill K: **Mass media interventions for smoking cessation in adults**. *Cochrane Database of Systematic Reviews* 2008, **Issue 1. Art. No.: CD004704. DOI: 10.1002/14651858.CD004704.pub2. Last assessed as up-to-date: 11 November 2007.**

26.Hey K, Perera R: **Quit and Win contests for smoking cessation**. *Cochrane Database of Systematic Reviews* 2007, **Issue 4. Update Feb 17, 2005**.

27. Hey K, Perera R: **Competitions and incentives for smoking cessation**. *Cochrane Database of Systematic Reviews* 2007, **Issue 4. Update: Feb 18, 2005**(4).

28. Sinclair HK, Bond CM, Stead LF: **Community pharmacy personnel interventions for smoking cessation**. *Cochrane Database of Systematic Reviews* 2004, **Issue 1. Art. No.: CD003698. DOI: 10.1002/14651858.CD003698.pub2.Last assessed as up-to-date: Oct. 30, 2008.**

29. Park EW, Schultz JK, Tudiver FG, Campbell T, Becker LA: **Enhancing partner support to improve smoking cessation**. *Cochrane Database of Systematic Reviews* 2004, **Issue 3. Art.No.:CD002928. DOI: 10.1002/14651858.CD002928.pub2. Last assessed as up-to-date: Feb. 24, 2008.**

30. Ussher MH, Taylor A, Faulkner G: **Exercise interventions for smoking cessation**. *Cochrane Database of Systematic Reviews* 2008, **Issue 4. Art. No.: CD002295. DOI: 10.1002/14651858.CD002295.pub3. Last assessed as up-to-date: July 5,2008.**

31. Stead LF, Lancaster T: **Group behaviour therapy programmes for smoking cessation**. *Cochrane Database of Systematic Reviews* 2005, **Issue 2. Art. No.: CD001007. DOI: 10.1002/14651858.CD001007.pub2. Date most recent amendment: Update: Feb 16, 2005.**

32. Lancaster T, Stead LF: **Self-help interventions for smoking cessation**. *Cochrane Database of Systematic Reviews* 2005, **Issue 3. Art. No.: CD001118. DOI: 10.1002/14651858.CD001118.pub2. Last assessed as up-to-date: 20 July 2005.**

33. Grimshaw GM, Stanton A: **Tobacco cessation interventions for young people**. *Cochrane Database of Systematic Reviews* 2006, **Issue 4. Art. No.: CD003289. DOI: 10.1002/14651858.CD003289.pub4. Last assessed as up-to-date: 18 October 2006.**

34. Lancaster T, Stead L: **Individual behavioural counselling for smoking cessation**. *Cochrane Database of Systematic Reviews* 2005, **Issue 2. Art. No.: CD001292. DOI: 10.1002/14651858.CD001292.pub2.Last assessed as up-to-date. Jul 14, 2008.**

35. EEbbert JO, Montori V, Vickers KS, Erwin PC, Dale LC, Stead LF: **Interventions for smokeless tobacco use cessation. Cochrane Database of Systematic Reviews**. *Cochrane Database of Systematic Reviews* 2007, **Issue 4. Art. No.: CD004306. DOI: 10.1002/14651858.CD004306.pub3. Last assessed as up-to-date: 17 October 2007.**

36. Hajek P, Stead LF, West R, Jarvis M, Lancaster T: **Relapse prevention interventions for smoking cessation**. *Cochrane Database of Systematic Reviews* 2005, **Issue 1. Art. No.: CD003999. DOI: 10.1002/14651858.CD003999.pub2 Last assessed as up-to-date: 24 January 2005.**

37. **Guide to Community Preventive Services. Decreasing tobacco use in worksite settings: incentives and competitions to increase smoking cessation among workers. www.thecommunityguide.org/tobacco/worksite/incentives.html <http://www.thecommunityguide.org/tobacco/worksite/incentives.html>. Accessed: July 16, 2009**

38. Moher M, Hey K, Lancaster T: **Workplace interventions for smoking cessation**. *Cochrane Database of Systematic Reviews* 2008, **Issue 4. Last assesed as Up-to-Date: Apr 23, 2008**.

39. Mazaik W, Ward K. D., Eissenberg T: **Interventions for waterpipe smoking cessation**. *Cochrane Database of Systematic Reviews* 2007, **Issue 4. Art. No.: CD005549. DOI: 10.1002/14651858.CD005549.pub2. Last assessed as up-to-date: 8 August 2007**.

40. Stead LF, Lancaster T: **Interventions to reduce harm from continued tobacco use**. *Cochrane Database of Systematic Reviews* 2007, **Issue 3. Art. No.: CD005231. DOI: 10.1002/14651858.CD005231.pub2 . Last assessed as up-to-date: 18 July 2007.**

41. Lovato C, Linn G, Stead LF, Best A: **Impact of tobacco advertising and promotion on increasing adolescent smoking behaviours**. *Cochrane Database of Systematic Reviews* 2003, **Issue 4. Art. No.: CD003439. DOI: 10.1002/14651858.CD003439.Last assessed as up-to-date: May 12, 2003.**

42. Repace J: **Benefits of smoke-free regulations in outdoor settings: beaches, golf courses, parks, patios, and in motor vehicles**. *William Mitchell Law Review* 2008:1621-1638.

43. Diethelm P, Rielle JC, McKee M: **The whole truth and nothing but the truth? The research that Phillip Morris doesn’t want you to see**. *Lancet* 2005, **366**:86-92.

44. Fiore MC, Jaיn CR, Baker TB, al. e: *Treating Tobacco Use and Dependence: 2008 Update. Clinical Practice Guideline. May 2008*. Rockville, MD: : U.S. Department of Health and Human Services. Public Health Service ; 2008.

45. **NICE  Guidelines on Smoking Cessation, Brief interventions and referral for smoking cessation in primary care and other settings. 3/2006. Australia - NSW Dep. Of Health 2005. www.nice.org.uk/page.aspx?o=SmokingCessationMain** [www.nice.org.uk/page.aspx?o=SmokingCessationMain]

46. Walters S, Wright J, Shegog R: **A review of computer and Internet-based interventions for smoking behavior**. *Addictive Behaviors* 2006, **31**(2):264-277.

47. Sargent J, Beach M, Adachi-Mejia A, Gibson J, Titus-Ernstoff L, Carusi C, et al.: **Exposure to Movie Smoking: Its Relation to Smoking Initiation Among US Adolescents.** *Pediatrics* 2005, **116**(5):1183-1191.

48. Tickle JJ, Sargent JD, Dalton MA, Beach ML, Heatherton TF: **Favourite movie stars, their tobacco use in contemporary movies, and its association with adolescent smoking.** *British Medical Journal* 2001, **10**:16.

49. Sargent J, Dalton M, Beach M: **Exposure to cigarette promotions and smoking uptake in adolescents: Evidence of a dose-response relation**. *Tob Control* 2000, **9**:163.

50. **National Cancer Institute. The Role of the Media in Promoting and Reducing Tobacco Use. Tobacco Control Monograph No. 19.Bethesda, MD:U.S. Department of Health and Human Services, National Institutes of Health, National Cancer Institute, NIH Pub.No. 07-6242, June 2008.**

51. Fichtenberg C, Glantz S: **Effect of smoke-free workplaces on smoking behavior: systematic review**. *British Medical Journal* 2002, **325**:188.

52. Aveyard P, Markham WA, Cheng KK: **A methodological and substantive review of the evidence that schools cause pupils to smoke**. *Social Science & Medicine* 2004, **58**(11):2253-2265.

53. Rosen L, Verbov G, Amitai Y, Stein-Zamir C, Knishkowy B: **Reaching Jewish Ultra-Orthodox Adolescents: Results from a Targeted Smoking Prevention Trial**. In *14th World Conference on Tobacco OR Health*. Mumbai; 2009.

54. Levine H, Borowski J, Bar-Zeev Y, Shreir E, Zarka S: **Smoking Prevention- What can a primary physician accomplish in his unit?** . *Journal of Israeli Military Medicine* 2007, **4**:202-204 (Hebrew)

55. **WHO Report on the Global Tobacco Epidemic, 2008. Fresh and Alive: MPOWER. Internet site: http://www.who.int/tobacco/mpower/mpower_report_full_2008.pdf.**
